# 加速康复外科从理论到实践——我们还需要做什么？

**DOI:** 10.3779/j.issn.1009-3419.2017.04.01

**Published:** 2017-04-20

**Authors:** 国卫 车, 伦旭 刘, 清华 周

**Affiliations:** 1 610041 成都，四川大学华西医院胸外科 Department of Thoracic Surgery, West China Hospital, Sichuan University, Chengdu 610041, China; 2 610042 成都，四川大学华西医院肺癌中心 Lung Cancer Center, West China Hospital, Sichuan University, Chengdu 610041, China

**Keywords:** 加速康复外科, ERAS方案, 可操作, 可评估, 可重复, Enhanced recovery after surgery, ERAS protocol, Operability, Evaluation, Repetition

## Abstract

加速康复外科（enhanced recovery after surgery, ERAS）的临床实践已有充分的证据改变了外科手术的结果，缩短住院日并节约费用。但是目前ERAS无论是被应用的广度还是深度却远远不够，原因何在呢？我们分析可能主要原因是缺少"可操作、可评估、可重复"的临床方案。可操作主要是指临床方案简单易行，团队和患者依从性均好；可评估是指方案应用前、中、后均有客观评估标准及处理方案；可重复是临床方案在本单位及推广过程中重复性好。

## Abstract (en)

Enhanced recovery after surgery (ERAS) is a paradigm shift in perioperative care, resulting in substantial improvements in clinical outcomes, shorter length of hospital stay and cost savings. But the current ERAS either by application of breadth or depth is not enough, why? The main reason is the lack of "operability, evaluation, repetition" ERAS protocol and suitable for clinical extensive application protocol. How to form the clinical available protocol? Operational mainly refers to the clinical scheme is simple and feasible, and protocol compliance is good; Evaluate refers to the methods used before, during and after are the objective evaluation criteria and plan; Repeatable is clinical scheme repeatability in the process of single or multiple center.

加速康复外科（enhanced recovery after surgery, ERAS）也有译为增强术后恢复计划（个人觉得这种翻译不易造成歧义），是一种多模式的围手术期综合诊疗路径。其目的是通过对手术进行风险评估和干预，优化治疗共存病症包括心血管、呼吸系统和/或肾脏疾病，同时治疗、维持患者在围手术期重要器官功能，了解和处理患者存在的社会和行为因素，比如对烟草和酒精的依赖等等，减少术中和术后患者身体对外科手术的严重应激反应^[1]^。实现途径有快通道麻醉（fast-track anesthesia, FTA）、微创外科技术（minimally invasive surgery, MIS）、最佳镇痛技术和强有力的术后护理（如术后早期进食、运动）等。因此，ERAS是新的外科理念，是将围手术期常规治疗措施进行优化和组合，达到降低并发症和病死率，缩短住院时间的目的，并非外科学的独立分支，而是对传统外科学的重要补充和完善^[2]^。这种“高大上”的理念为何难以“接地气”呢？本文将围绕胸外科围手术期加速肺康复临床应用实践，从临床方案的“可操作，可评估，可重复”进行论述和交流。

## 1 “可操作”的临床方案是ERAS开展的基础

可操作的临床方案主要包括三方面的含义：①方案本身简单、易行，各个环节均易操作，主要是不能增加工作量；②团队各个成员均既了解全过程又能完成自己工作，“承接和下传”均有效，一个环节出问题可能整个方案均失败；③医、护、患的依从性高，医生和护士通过方案实施，提高业务水平和个人职业生涯；患者能够加快术后康复，减少痛苦和节约费用，双方均满意。如何才能同时做到这几点呢？我们认为应该是以“病人为中心”和“问题为导向”（图 1），以解决科室管理和术后患者常见和共性问题为主。以胸外科肺部手术为例，术后最常见和最严重并发症是肺部感染，而肺部感染的影响因素贯穿围手术期，涉及多个专业，因此需要多个学科相互协作。问题是目前肺部术后肺部感染发生率很低，若用统一的加速肺康复方案，其实是增加方案执行难度，因为针对解决肺术后肺部感染的方案有其特定的人群（如高龄或肺功能差、及手术复杂的患者），而对年轻及手术相对简单患者，很多操作过程是没必要的，实际是增加了操作的难度。这个问题如何解决呢？仍然坚持问题导向，目标管理。如针对肺部手术术前存在高危因素患者，以降低术后并发症和死亡率为目标，核心任务是肺康复训练（图 2）^[3-6]^。ERAS团队需要康复科（术前肺功能评估与肺康复训练，术后康复训练等）、麻醉科（针对合并症及手术难度，采用不同麻醉方法，合理镇痛等）、胸外科（最佳手术方案，如微创等）、重症监护室（通气方案及预防血栓等）和护理（合理饮食及运动方案等）。而无高危因素患者，以提高住院舒适度和缩短住院时间为目标，核心任务是围手术期流程优化^[7-10]^。ERAS团队胸外科（微创手术方案及缩短手术时间^[11]^），麻醉科（不插管麻醉，预镇疼等），手术室（尿管是否可以不用或术中安装，清醒时去除^[12-15]^（图 3）；引流管可以不用或1根或细引流管等^[16-19]^），病房护士（术后病房不用心电监护，饮食管理^[13]^及早期下床活动等）。对于术后出现不需要住院处理，需要观察处理或服用药物治疗的症状患者，以控制症状、改善生活质量为目标，核心任务是症状管理。ERAS团队组成主要是胸外科医生（解释及心理）护士（随访及解释），康复科（家庭肺康复训练方案及指导）或呼吸科（咳嗽及对症处理），中西医结合科（腹胀等症状调理）。针对患者所有症状我们都能向患者提供相应治疗方案并辅以心理疏导，才能提高医患双方的依从性。医护依从性提高主要是团队相互协调和支持，患者依从性的前提是住院舒适度和机体快速康复。

**1:**
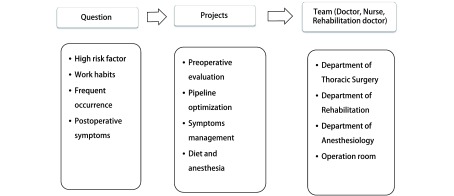
问题导向的ERAS研究方案 The ERAS protocol established from question. ERAS: enhanced recovery after surgery

**2:**
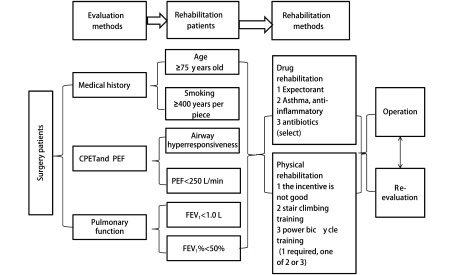
肺康复方案及流程（四川大学华西医院胸外科） Pulmonary rehabilitation scheme and process (Department of Thoracic Surgery, West China Hospital, Sichuan University). CPET: cardio-pulmonary exercise test; PEF: peak expiratory flow; FEV_1_: forced expiratory volume in one second.

**3:**
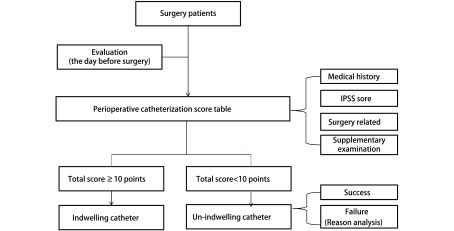
ERAS方案流程优化之尿管管理 Optimization of catheter management in ERAS program

可操作方案均需要团队协作与配合，多模式医疗（multimodal perioperative care）和多学科协作（multidisciplinary team approach），何种模式更有利于ERAS顺利实施呢？以外科医生或技术为主的多模式是早期外科快速康复实践中的主要手段，外科医生为主导，麻醉师，康复师或护士提供方案，最后在外科医生的指导下实施，如术前肺康复训练方案及周期，基于微创技术的流程优化。此种模式的最大优点是易于操作，方案固定，所有执行人员都有章可循。ERAS多模式医疗可能主要适用于选择的病种或病例，ERAS方案相对简单、易行^[14]^，如疼痛管理，外科医生负责区域阻滞，麻醉医生关注全身用药和副作用，护理则适时进行评估并反馈结果^[15]^。加速康复外科领域的扩展和深入，外科为主导的多模式医疗方法实现难度不断增加，麻醉医生为主“围手术期外科之家（perioperative surgical home）”的多模式是一种探索，在康复团队中扩大麻醉医生作用和工作范围（主导作用），麻醉医生参与术前评估，术中合适麻醉方法的选择及ICU管理，全程管理、记录和评价方案效果，有助于积累经验和方案的持续改进^[16]^。多模式医护方案应用于临床研究或规模比较小的医院可能有其现实性，但是对于多中心临床研究或推广则需要多学科的协作。多学科协作模式有助于安全性和达到共识并推广，这需要团队先制定某个病种快速康复目标，达成共识，然后大家优化方案并执行，记录结果与优化。但是多学科协作的主要不足是每个专科会过多的将过于专业的方案纳入ERAS总体方案，使方案繁锁而难以实施^[17]^。如何使学科之间围绕ERAS进行深度融合是研究的方向。

如何以“病人为中心”，打破科室之间的“围墙”，简化流程和步骤，均需要学科协作和医护一体。华西医院胸外科早期的方法是以问题立项目，以项目建团队，共同参与。项目完成时，大家的认识在提高的基础上形成共识，并逐步推广应用。

## 2 “可评估”的临床方案是ERAS顺利实施的保障

ERAS临床方案的“可评估”性主要体现在以下几方面：①基于患者“个体化”ERAS方案的制订，合理的评估方法；②ERAS方案在各个团队执行过程中，使每个环节都有客观评估体系，使每个过程都达到目标；③针对每个ERAS方案全程效果评估，再达到优化目标。

首先仍需针对现有ERAS评估标准进行分析，加速康复外科的实质是降低医疗应激反应（手术及治疗创伤），机体生理功能快速恢复。而其临床实现或体现需要判定标准，统一评价标准是ERAS临床获得循证医学证据方案所必需。当前作为评价ERAS方案可行与否的标准，应用最多的是降低术后并发症和缩短住院时间，这是从“医生角度”进行评价^[1]^；准确反映患者机体状况和感受，而提出症状恢复（patient-reported outcomes, PROs）作为评定是否快速康复标准^[25]^，是从“患者角度”进行评价。强调住院日缩短和费用降低作为判断ERAS方案是否成功的标准，是从“社会角度”^[1]^。通过对中国大陆胸外科医护问卷调查发现，将三者联合起来作为“ERAS方案执行成功与否”的标准得到大家认可^[26]^。

其次我们仍然以肺部手术ERAS方案制订看“可评估”性。每一位肺部疾病患者术前都根据病史、肺功能（pulmonary function test, PFT）、心肺运动试验（cardiopulmonary exercise testing, CPET）及呼吸峰值流速（peak expiratory flow, PFE）进行高危因素评定，然后制定“个体化方案”（图 3、图 4）。高危因素不同的患者应用不同的术前肺康复训练方案，疗程结束后，进行评估是否达到手术标准，否则再进行一个疗程肺康复训练，最多三个疗程。这个评估标准和训练方案，团队成员人人知晓，患者从训练到评估，人人清楚，所有人员都可监督患者执行和随时评估^[27, 28]^。非高危因素患者，主要是流程优化，所有需要优化的流程也有相应的评估体系：如尿管应用，病房护士对每一位患者术前应用评估表（分值决定是否留置）进行评估，并决定是否应用^[13]^；若应用，进行宣教及术前、术后注意事项。麻醉师会相应控制术中输液量，手术室护士会术中会评估膀胱充盈度，根据情况随时决定是否需要重新安置尿管^[15]^。术后回到病房护士会对膀胱充盈程度评估，并应用鼓励或诱导（卫生间站立排尿或水龙头滴水等）尽量自行排尿^[5]^。胸腔引流管应用1根或2根，管径粗细均应术前评估^[17]^，术后有观察评估体系。若有失败环节，并做好记录，分析原因，进一步完善或修正相应评估表格。

**4:**
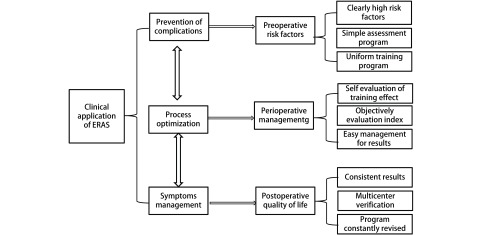
“个体化”ERAS方案可评估性 "Individualized" ERAS program and evaluation

最后这种“可评估”的表格及措施，①使ERAS方案的可操作性更强，每个成员都很清楚自己工作的效果及评价方法；②处处和随时评估，有助于及时发现并纠正问题，保障医护患者安全，也增加了可操作性；③及时评估并发现问题，有助于不断纠正ERAS方案，使其更加切合临床实际，确保ERAS方案实施的可持续性和安全性。

## 3 “可重复”的临床方案是ERAS普及推广的前提

只有得到循证医学证实和临床重复应用均达到目标的ERAS方案才具有生命力和造福患者。ERAS方案的“可重复”性主要体现在：①针对某医院，某个医疗组（或团队），某病种具有可重复性，也是“个体化”之一，需要进一步优化；②针对特定级别医院（如三级甲等医院），某病种具有可重复性，这些方案有一定的推广价值；③针对某病种，所有医院都可应用的方案。可重复也意味着我们不能照搬照抄现有的方案，若不结合单位和个人实际，可能会造成相反的结果。现在临床ERAS方案推广难的主要原因也是我们在应用某些方案时，理解有偏差，单位条件不具备时，盲目应用而导致效果差，从而体会不到加速康复的优势而放弃应用。

如何才能增加ERAS方案临床应用中的“可重复”性呢？首先是加强ERAS临床方案和宣传，包括团队中的每个成员和患者，有研究^[29]^表明每周时间内周一、周二应用效果优于周四、周五，与方案实施中人员周末不在有关；其次是不同单位及不同团队应用时都要根据情况对方案进行修正，符合实际情况，并建立临床项目，合理评估每个环节并修正之；再次ERAS方案要进行多中心研究（要体现医院区域性及差异性，如教学医院，省、市级和县医院）。最后ERAS方案实施过程中同一操作要有多种方法备选（如肺康复训练时，需心肺运动专用设备，若没有可选登楼运动，我们的工作是要将专业设备训练指标与登楼运动的等效指标提出来^[30]^），这样不同医院可重复性会更强，ERAS才会从“高大上”到“接地气”。

## 4 ERAS方案的“高大上”如何才能“接地气”

ERAS方案作为主体实施者医护依从性差为何不高呢？分析主要原因可能有：①ERAS方案临床应用效果不明显，不顾条件盲目套用；②住院日没有缩短和缩短后再入院率高，执行过程中评估体系差，没有及时发现和处理问题。③术后并发症（术后恶心、呕吐、疼痛和肺部感染）也是依从性逐渐性降低的因素之一，即使在大的医学中心也是如此。④术前具有高危因素的患者进行ERAS程序导致失败而产生放大的“安全性”顾虑。⑤缺乏有效的、大规模临床试验并得到好的ERAS方案进行推广；⑥术后及出院症状管理随访管理不完善，导致患者满意度低。也是加速康复外科的主要组成部分，从肺癌患者出院后的主要症状并分析其原因，优化手术方案、围手术期管理流程及合理的出院后管理，会促进加速康复外科推广及临床应用。

如何增加ERAS方案的依从性呢？①方案的早期实施阶段应加强对团队成员专业训练，结果的持续性评估，方案的依从性在早期是会降低的。②医生要坚持应用并总结经验。③降低术后并发症也是重要手段之一，多中心研究发现并发症的降低与ERAS依从性呈正相关。④团队合作与质量持续改善计划，团队制订ERAS方案和目标管理，如住院时间达到多少等，并持续坚持、学习总结策略。⑤多模式或多学科协作，术前重视患者教育、沟通与合作是成功的基础。⑥术前高危因素患者评估、准备及治疗，降低ERAS方案失败率也是增加依从性的主要措施。⑦国际协会和专业协会推荐与推广，这需要严格具有循证医学证据的临床研究。

如何才能形成这样的方案呢：我们的经验是从临床问题立项目，项目建团队，团队求方案，多中心找证据。“协同创新、学科协作、医护一体”，以临床与科研相结合，最终形成“可操作、可评估、可重复”简单、易行的基于病种的ERAS方案（图 5）。

**5:**
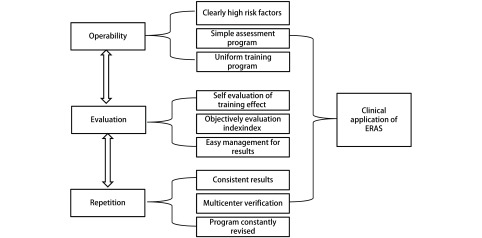
ERAS方案的形成与验证流程 The formation and validation of the ERAS program

总之，我们认为微创外科及其体系完善是加速康复外科发展的动力，医护一体和多学科协作是加速康复外科顺利实施的保障，加速康复外科临床方案的规范化应用必将造福患者。加速康复外科从理念到实践的实施，必将达到“让手术不再痛苦，让患者不再害怕手术”的愿景。
